# Adult childhood cancer survivors’ perceptions of factors that influence their ability to be physically active

**DOI:** 10.1007/s00520-023-07865-6

**Published:** 2023-06-22

**Authors:** Laura Jess, Maria Bäck, Marianne Jarfelt

**Affiliations:** 1 Department of Oncology, Institute of Clinical Sciences, Sahlgrenska Academy, University of Gothenburg, Gothenburg, Sweden; 2 Närhälsan Bollebygd Rehabilitation Center, Bollebygd, Sweden; 3 Department of Occupational Therapy and Physiotherapy, Sahlgrenska University Hospital, Gothenburg, Sweden; 4 Department of Health, Medicine and Caring Sciences, Division of Prevention, Rehabilitation and Community Medicine, Unit of Physiotherapy, Linköping University, Linköping, Sweden; 5 Department of Molecular and Clinical Medicine, Institute of Medicine, Sahlgrenska Academy, University of Gothenburg, Gothenburg, Sweden; 6 The Long-Term Follow-up Clinic for Adult Childhood Cancer Survivors and Cancer Rehabilitation, Sahlgrenska University Hospital, Gothenburg, Sweden

**Keywords:** Content analysis, Exercise, Late effects, Pediatric cancer survivorship, Qualitative research

## Abstract

**Purpose:**

Studies indicate that adult childhood cancer survivors do not achieve recommended physical activity levels. A deeper understanding of factors that influence their ability to be physically active is essential to identify individuals in need of support. The aim was to explore factors that influence adult childhood cancer survivor’s ability to be physically active.

**Method:**

Semi-structured interviews were conducted from June to October 2020 with 20 adult childhood cancer survivors with a median age of 31 (min–max 20–47) years. Interviews were transcribed verbatim and analyzed with qualitative content analysis.

**Results:**

Four main categories: “The impact of environmental factors,” “Personal factors of importance,” “Consequences of the treatment or disease,” and “The impact of support from healthcare” and 10 sub-categories, were identified. Participants described how family habits and encouragement from others influenced their present ability to be physically active. Experienced benefits of physical activity were described as a facilitator for current physical activity while suffering from late complications was identified as a barrier. Participants highlighted the importance of specific and individualized physical activity recommendations.

**Conclusion:**

This study includes adult childhood cancer survivors several years after completion of treatment, hence highlighting the importance for support both during treatment and follow-up to sustain their physical activity. Healthcare providers need to identify individuals suffering from late complications, even several years after treatment; provide individualized physical activity recommendations; and educate families and schools about the importance of physical activity in childhood cancer survivorship.

**Trial registration:**

This research project was registered in the Swedish National Database of Research and Development, identifier 273320, December 6, 2019 (https://www.researchweb.org/is/vgr/project/273320)

**Supplementary Information:**

The online version contains supplementary material available at 10.1007/s00520-023-07865-6.

## Background

Due to the risk of late complications and increased health problems, it is important for adult childhood cancer survivors (ACCS) to follow healthy lifestyle behaviors, including regular physical activity [1]. The number of ACCS is increasing due to a high survival rate [2, 3]. However, 60–80% suffer from complications from the disease and the treatment over time [4–6]. These include different types of physical (including organic diseases), cognitive, and psychological consequences depending on type of cancer, treatment, and age at treatment [4–8]. Currently, it is recommended for ACCS to follow the same physical activity guidelines as the general population [9], including ≥ 150-min moderate-intensity cardiorespiratory physical activity or ≥ 75 min of vigorous-intensity cardiorespiratory physical activity or a combination thereof throughout the week. This should be combined with strength exercises ≥ 2 days a week and a reduced amount of sedentary time [10]. Previous studies indicate that ACCS do not achieve sufficient physical activity levels and have poor levels of physical fitness [11–13].

Qualitative research can be a tool in gaining a deeper understanding of factors that might impact on ACCS’ ability to be physically active. Recently, two reviews [14, 15] identified late complications of childhood cancer and its treatment such as loss of strength, pain, psychological barriers, and lack of social support, as common barriers to being physically active. However, most of the studies are performed during treatment or with short follow-up. Only two of the studies included in the reviews adopted a purely qualitative approach and had a particular focus on barriers and facilitators to physical activity in ACCS > 18 years [16, 17]. Therefore, increased in-depth knowledge about ACCS’s perceptions of factors that influence their ability to be physically active is needed to identify individuals in need of support to maintain or increase their physical activity level. The aim of this study was to explore factors that influence ACCS’ ability to be physically active.

## Methods

### Design

Data is reported in accordance with the COnsolidated criteria for REporting Qualitative research (COREQ) [18].

### Participants

ACCS were identified at the long-term follow-up clinic (LTFU) at the Department of Oncology, Sahlgrenska University Hospital, Gothenburg, Sweden. Patients were eligible if they were aged ≥ 18 years, were diagnosed with cancer at < 18 years of age, were at least 5 years post treatment at the time of study, and had attended the LTFU within the last 18 months. Exclusion criteria were ongoing recurrence or second malignancy, impaired cognitive or communicative ability, or insufficient knowledge of the Swedish language. A strategic sampling strategy was used including childhood cancer diagnoses, gender, age when diagnosed with cancer, and age at study inclusion.

A total of 26 ACCS were invited, of whom 20 accepted to participate. Reasons for declining participation were lack of time (*n* = 3) or not specified (*n* = 3). Demographic data of the participants are stated in Table 1.

**Table 1:** Demographic and clinical characteristics of the study population

Adult childhood cancer survivors	*n* = 20	%
Men	11	55
Age at diagnosis, years, median (min–max)	8.5 (1–15)	
Age at study, years, median (min–max)	28.5 (18–51)	
Years since diagnosis, median (min–max)	23 (11–38)	
Highest level of education		
Upper secondary school	11	55
University degree	8	40
Home situation		
Married/co-habitation	8	40
Living alone	8	40
Living with parents	4	20
Diagnosis		
Leukaemia (ALL, AML)	6	30
Lymphoma (HL, NHL)	3	15
Sarcoma (bone or soft tissue)	3	15
Malignant brain tumour	4	20
Spinal tumour	1	5
Wilms tumour	2	10
Neuroblastoma	1	5
Treatment		
Chemotherapy	19	95
Surgery	10	50
Radiation	11	55
Chemotherapy + radiation	2	10
Chemotherapy + surgery	4	20
Surgery + radiation	5	25
Surgery + chemotherapy + radiation	5	25

Except for age and years since diagnosis, data is presented in numbers *n* and percent (%)

*ALL* acute lymphoblastic leukaemia, *AML* acute myeloid leukaemia, *HL* Hodgkin’s lymphoma, *NHL* non-Hodgkin’s lymphoma

### Setting

The setting is at the LTFU for ACCS at Sahlgrenska University Hospital. ACCS are referred from pediatric cancer care, from other specialties, or by contacting the LTFU themselves. Most of the participants did not have any or limited follow-up from healthcare during adulthood before their visit to the LTFU. In Sweden, healthcare including physiotherapy is funded through the national healthcare system. However, no physiotherapists are yet employed at the six established LTFU in Sweden.

### Procedure

Twenty interviews were conducted from June until October 2020. MJ identified eligible patients. An introductory letter with brief information about the study was sent and followed up with a telephone call. Individuals who were interested in participation received detailed written and oral information. Written informed consent was obtained prior to the interview. Due to the COVID-19 pandemic, interviews were performed either by a digital meeting (*n* = 19) or by phone (*n* = 1), as a self-preferred choice. A pilot interview was performed prior to the study to test the interview guide. A question about their family’s attitude towards physical activity was added and the pilot interview was included in the analysis. All interviews were performed by LJ and started with the question “What do you think of when I say physical activity.” The interviews were semi-structured and based on an interview guide (Appendix 1). Follow-up questions were used to deepen the dialog. The interviews were recorded with an Olympus Digital Voice Recorder and lasted for a median of 28 min (min 16–max 58).

### Analysis

The interviews were transcribed verbatim and analyzed using qualitative content analysis by Graneheim and colleagues [19]. To enhance trustworthiness and credibility, the data analysis procedure was planned a priori. First, the interviews were read several times to get a sense of the whole. Then, meaning units were constructed to shorten the text without losing the core intent. The meaning units were labelled with a code and codes with similar content were arranged in sub-categories and categories (see Table 2).

**Table 2:** Example on the content analysis

Meaning unit	Condensed meaning unit	Code	Subcategory	Category
I get happier, I have a more general positive attitude. I sleep better, I can also notice that on days when I have not activated myself physically, it can be more difficult to fall asleep because I am not tired in the same way [19]	Happier, more general positive attitude, better sleep Days without physical activity harder to fall asleep, not as tired	Positive experiences influencing physical activity	Previous experiences influencing current physical activity	Personal factors of importance for being physically active

An analysis of the first steps until creating codes was performed by LJ. A second analysis, including the same steps, was conducted by MB on the first five interviews and the results were compared. Finally, MJ joined the analysis and all the authors worked together to define sub-categories and categories and a theme until consensus was achieved. This stage was an iterative process, which was refined at several meetings. LJ is a physiotherapist and PhD student with clinical experience of adult cancer rehabilitation. MB is a physiotherapist and associate professor with skills in qualitative research methodology and in-depth knowledge of the construct physical activity. MJ is a senior consultant at the LTFU and an associate professor in late complications of childhood cancer.

## Results

**Fig. 1:**
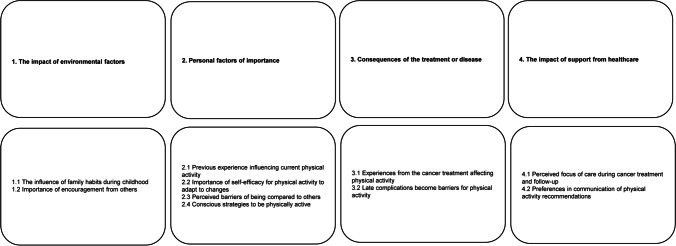
An overview of the categories and sub-categories

The analysis resulted in four main categories and 10 sub-categories (see Fig. 1).

### The impact of environmental factors (category)

Participants described how family habits and encouragement from others had a big influence on their earlier and current ability to be physically active.

#### The influence of family habits during childhood (sub-category)

Participants expressed how growing up in a family where physical activity was a natural habit was a facilitator for continuous physical activity. When parents, siblings, or grandparents engaged in physical activities, they acted as important role models.

“I have a good example in my family with my grandfather who is very active, he has exercised throughout his life and taken very good care of his body.” (Participant 16)

In contrast, it was a perceived barrier to grow up in a family which did not prioritize sport activities. To start a new sport as an adult, when missing the experience from childhood, was described as difficult.

“I still feel that it is difficult to participate in physical activities, because I do not have the same previous experience as others regarding physical activity and different sport activities.” (Participant 6)

#### Importance of encouragement from others (sub-category)

Participants stated encouragement from others as key factor for being physically active in their childhood, which also influenced their present situation. Support might be described as encouragement to try out new activities or to continue with activities during treatment. Lack of parental encouragement was perceived as a barrier to being physically active and retaining a physically active lifestyle.

“My parents have always encouraged me to be physically active … But they even listened when I said I can’t manage it. So it’s been positive in every way…” (Participant 14)

Participants identified encouragement from teachers in school as an important factor. Teachers who suggested alternative exercises during and after cancer treatment influenced participant’s possibilities to continue sports in school.

“…But I believe in a collaboration with the school … to get help to find activities ... What works instead?” (Participant 1)

Participants who at an adult age had experiences of exercising with other ACCS through a patient organization, described this as undemanding and felt encouraged. However, some participants expressed frustration because exercises still were too difficult.

“When you have experiences throughout your life, I’m not able to do what others do and then suddenly when you're in that group where it’s really supposed to be inclusive… then there is kind of a double exclusion in here I don’t fit in either.” (Participant 6)

### Personal factors of importance (category)

Participants’ attitudes towards physical activity were influenced by personal factors as previous experiences of physical activity, self-efficacy, comparison with others, and conscious strategies.

#### Previous experiences influencing current physical activity (sub-category)

Previous experienced benefits of physical activity were described as a facilitator for participants’ present physical activity. These benefits included physical, psychological, and social aspects, such as reduced pain, increased self-efficacy, and social interactions. Physical inactivity could be associated with decreased mental-health and impaired sleep.

“For me, it is very important for my mental well-being… I really experience that if I am not physically active so… then I get negative thoughts.” (Participant 7)

Negative experiences or associations with physical activity were identified as a barrier. These included relating physical activity to anxiety, bullying, and boredom.

#### Importance of self-efficacy for physical activity to adapt to changes (sub-category)

Self-efficacy for physical activity was identified as an important factor. Low self-efficacy was described as having low confidence in one’s ability to be physically active and perceived difficulties in adapting to individual abilities.

“It’s a lot about being able to adjust and finding types of exercises that work for me. It’s probably that I have to work a lot with myself and practice accepting ‘I can’t do this, but I can do this instead…’” (Participant 17)

#### Perceived barriers of being compared to others (sub-category)

Some participants reported that they avoid social media or do not want to exercise with others, because they feel uncomfortable due to bodily changes. Continuing with team sports in adolescence, when the focus changed from being recreational to competitive, was experienced as difficult.

“That I don’t want to exercise with others, has to do with the fact that I’m insecure in myself... and this has affected me a lot.” (Participant 4)

#### Conscious strategies to be physically active (sub-category)

Participants had different conscious strategies to be physically active. These were described as exercising directly after work, goal setting, and using different digital tools. Making physical activity a habit and part of the daily routine was described as crucial. Participants expressed the importance of choosing activities which feel meaningful or fun, but said they sometimes exercise even if they did not feel like it.

“… I reason that I at least can go there to stretch a bit or go there to practice handstands, because that is fun and more like a plaything… and when I get there and warm up, suddenly, I get motivated and do a usual exercise session anyway.” (Participant 2)

### Consequences of the treatment or disease (category)

Participants sometimes described how symptoms due to cancer treatment or late complications affected their ability to be physically active both during cancer care and in their present situation.

#### Experiences from the cancer treatment affecting physical activity (sub-category)

Apart from specific restrictions after surgery or due to neutropenia, the participants described physical limitations as nausea, feeling sick, pain, and balance problems as reasons for not being able to be physically active at school or leisure time.

“… but since the treatment affected my balance, I did not learn to ride a bike and skate and all this until several years after everyone else in school.” (Participant 9)

Some participants recalled how they had to stay in bed during the treatment period, while other participants described how they were able to continue with their sport activities if their blood levels were acceptable.

“… as long as my blood values allowed me to interact with other people, I could still exercise. I played handball, even during treatment.” (Participant 5)

Not being able to attend sports activities during treatment resulted in different consequences which some described as missing out on friendship and training camps or being forced to quit their sport.

“… that was when I had to sell my horse, because I was not allowed to be in the stable …due to… that I had a weakened immune system… so it became an unsustainable situation. It did not work.” (Participant 4)

#### Late complications become barriers for physical activity (sub-category)

Suffering from physical or psychological late complications was identified as a current barrier for being physically active. Participants described how some of the symptoms appeared during cancer treatment and gradually got better, while others experienced new symptoms many years after completion of treatment. However, some participants did not experience that their ability to be physically active was affected due to late complications.

“I was very active as a child… but it was the disease and everything that came later that made me quit.” (Participant 11)

Late complications could include orthopedic complications causing pain and reduced function, balance problems, breathing issues, and fatigue. Some participants described psychological late complications affecting their physical activity participation, including fear of injury and being overly cautious.

“I was very sensitive to what the body felt… at the slightest sensation… now something is wrong, now I got injured… I think my nervousness went to exaggeration.” (Participant 18)

### The impact of support from healthcare (category)

Participants had different experiences regarding support from the healthcare including receiving physical activity recommendations. The healthcare provider’s ability to meet their specific needs was described as important for their present ability to be physically active.

#### Perceived focus of care during cancer treatment and follow-up (sub-category)

Participants experienced major differences between cancer treatment and the follow-up care. The cancer care was described as fantastic, while participants tended to feel that it was up to themselves to get a follow-up. This included help regarding late complications or specific physical activity advice. Some participants criticized how the check-up only focused on the medical part and not the whole person.

“My image of Swedish healthcare is that it is fantastic if there is something urgent and then you are left completely alone.” (Participant 11)

“It is a trauma in itself to get cancer. Taking care of the whole person and not just the medical part would be great.” (Participant 5)

Participants also described difficulties in identifying if physical impairments were related to normal aging, typical sport injuries, or late complications. Having questions where the knowledge is limited today was described as challenging.

“It is difficult when one’s symptoms are not treated as late complication, but almost ‘this cannot be true because the research has not provided it yet’” (Participant 6)

#### Preferences in communication of physical activity recommendations (sub-category)

Participants experienced physical activity recommendations as too general which made them hard to apply. General information instead of specific in-depth recommendations was perceived as a duty rather than helpful and could result in reduced motivation. Furthermore, participants highlighted the importance of giving physical activity recommendations in a supportive way without blaming.

“You need a little more in-depth advice about the whole thing, instead of feeling like a requirement that I should be physically active.” (Participant 6)

Some participants expressed a need for specific physical activity recommendations but also clear restrictions to prevent them making up their own restrictions after cancer treatment.

“I think that when you have been through such a tough treatment and very ill, you may become a little afraid of the body’s signals and what you can expect if you exercise. It’s probably good to have guidance in how to start rebuilding the body.” (Participant 15)

Participants could report that physical activity recommendations during treatment were not feasible due to their young age and since the focus was on surviving. However, some participants perceived early information about the risk for comorbidities due to inactivity as a facilitator for developing a healthy lifestyle. Participants could also criticize how the healthcare providers only talked to the parents instead of involving them.

“… when I finished treatment or during the treatment, the last thing I wanted to think about was exercise. It was not interesting to me at all. The only thing I wanted to focus on was to continue breathing and not to die.” (Participant 18)

“But I experienced that people were not good at talking to me at all during treatment, they talked more with the parents during that time.” (Participant 5)

Physiotherapy was sometimes associated with boredom and participants expressed a wish for support in finding alternative exercises that could be applied outside the hospital.

“The problem for me was that physiotherapy felt so boring… I did not get the same feeling you get after a good workout… therefore I didn’t have not the motivation to continue.” (Participant 18)

## Discussion

This study adds in-depth knowledge about ACCS’ perceptions of factors that influence their ability to be physically active several years after completion of treatment. These aspects include environmental factors, personal factors, consequences of the treatment or disease, and support from the healthcare provider. There is a growing population of ACCS, but unlike our study, previous research on motivation for and potential barriers to physical activity has mainly been performed during treatment or with short follow-up. Increased knowledge about perceived barriers to physical activity is essential for healthcare providers in pediatric oncology and in follow-up care when developing strategies and improving routines to support ACCS to overcome these specific barriers.

Our study illustrates the diversity among ACCS. Some of the participants did not suffer from late complications, while other participants were struggling with both physical and psychological late complications which impaired their ability to be physically active. This finding has been reported in research of adult cancer survivors where treatment-related side effects and kinesiophobia were common barriers for physical activity participation [20]. To overcome these barriers, healthcare providers could educate ACCS about their condition and how physical activity can improve their symptoms. This study highlights the need for professionals with the competences to identify individuals in need of support to increase their physical activity even for a long time after completion of treatment, in the LTFU that now are being established in many countries.

Participants wish to receive meaningful exercises from the physiotherapist that can be applied outside the hospital; this is similar to the results from a review of survivors of adult cancer that exercise recommendations in secondary prevention must be more specific regarding exercise dose and have to be based on the individual ability [21]. The importance of timing and need of age-appropriate adaptation when giving information about physical activity recommendations is in line with a recently published review which found that there were different barriers and facilitators to physical activity participation depending on age and life stage [15]. However, in recent years, physical activity programs in pediatric oncology have been developed in many countries and the prerequisites for future ACCS may already have improved [22]. Some participants in our study described difficulty in assimilating advice about physical activity during and shortly after treatment since their focus was on surviving, while other participants appreciated information at an early age which helped to develop a healthy lifestyle. Prior research has shown significant knowledge gaps regarding late complications and health risks among ACCS, which seem to appear due to young age at diagnosis which can result in low level of responsibility for health self-management [23, 24]. This indicates the importance of balancing information when focus is on survival, with the need for information to increase health self-management. To balance information, recommendations about physical activity could be repeated at follow-up both during adolescence and adulthood. Thus, adequate transition into adult healthcare is essential.

Our results indicate that individual factors including previous positive experiences of physical activity and high self-efficacy may influence the ability to be physically active. In adult cancer survivors, perceived health benefits and positive previous experience of physical activity, especially in relation to improvement in cancer symptoms, were strong facilitators for being physically active, whereas low self-efficacy was a common barrier [20]. Comparing themselves to peers and siblings was described as a barrier in our study. Feeling uncomfortable due to bodily changes was one reason mentioned, which has earlier been shown in ACCS [25]. However, a study by Burke et al. [26] showed that ACCS were nervous about engaging in physical activity after cancer treatment but that self-efficacy and confidence increased after participation. Nevertheless, ACCS may need individualized support and guidance, and a combined program with physical and psychological support could be suitable to overcome these barriers. In the past years, several countries have increasingly implemented physical activity programs in pediatric oncology [22].

Participants in our study highlighted the importance of family habits and social support affecting their ability to be physical active, which is consistent with previous research on ACCS [27]. Studies of the general population emphasized the importance of early exposure to different sport activities and indicate that physical activity which becomes habitual in childhood may persist throughout adolescence and in adult life [28]. Research in parents to children with cancer indicates lack of awareness regarding the importance of physical activity in their child’s cancer survivorship, although they have knowledge of general benefits of physical activity [29]. This highlights the importance to increase the awareness in healthcare providers about this lack of knowledge about physical activity in cancer survivorship. Healthcare providers should involve families and schools of ACCS when developing strategies to overcome potential barriers for physical activity. Some interventional studies involving support from peers and parents in physical activity during hospitalization have already been performed [30, 31].

**Fig. 2:**
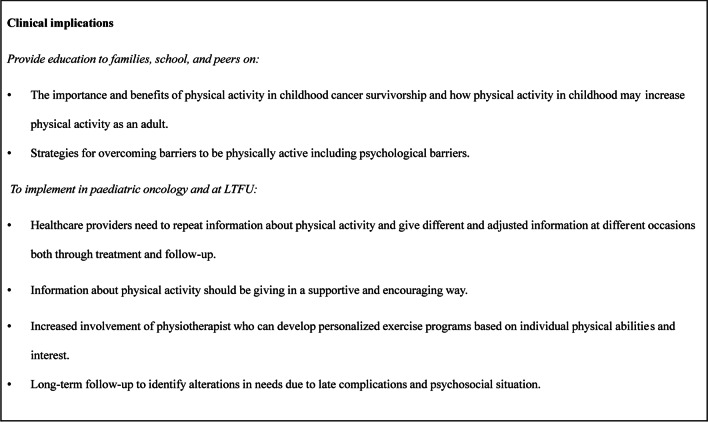
Clinical implications

Suggestions for clinical implications are stated in Fig. 2.

### Strength and limitations

In order to increase trustworthiness, participants of various ages, gender, diagnosis, and treatments were included. However, there may also be a need to study specific groups of patients. This study is unique as it includes ACCS several years after treatment. Nevertheless, this may also have increased the risk of recall bias due to time since treatment.

Another limitation is that transcripts were not returned to the participants for comments, which could have increased the credibility of results [32]. However, to increase credibility, quotations from the transcribed text are presented in the “Results” section and during the analysis, investigator triangulations were used until consensus was reached.

## Conclusion

This study includes ACCS several years after completion of treatment and highlights the need for support both during treatment and follow-up to sustain ACCS physical activity. Furthermore, our study emphasizes the importance of identifying ACCS who suffer from or are at risk for late complications even several years after treatment, as they need extended support to be physically active. Physical activity recommendations should be repeated and given in a specific, individualized, and supportive way. Findings in this study highlight how family habits, role models, and social support during childhood still play a crucial role in ACCS’ ability to be physically active in adulthood. Future interventions should involve the families, peers, and schools to improve their awareness about the importance of physical activity in childhood cancer survivorship aiming to build good physical activity habits in childhood and adolescence.

## Supplementary information

ESM 1

## Data Availability

The participants consent does not cover the distribution of the interview material or the transcripts.

## Abbreviations

LTFU: Long-term follow-up clinic
ACCS: Adult childhood cancer survivors
