# Supplementary material for: Adult childhood cancer survivors’ perceptions of factors that influence their ability to be physically active
Source: Support Care Cancer. 2023 Jun 22;31(7):409. doi: 10.1007/s00520-023-07865-6 (PMC10287765; doi:10.1007/s00520-023-07865-6)
Supplement: Supplementary file 1 — ESM 1 [file 520_2023_7865_MOESM1_ESM.doc]

1. What are you thinking of when I say physical activity
2. What are you thinking of when I say exercise?
3. Can you give some examples of daily activities that you perform a regular day?
4. Do you think there is a different between physical activity and exercise?
5. Can you describe your experiences of physical activity?
6. Did you exercise in childhood?
7. Do you experience any physical hinder that affect your ability to be physically active?
8. Do you experience that the treatment you received during childhood affect your ability to be physical active?
9. Physical activity, do you think it is important?
10. Did you get any recommendations or support regarding physical activity during or after you were treated for cancer in your childhood?
11. Have you any experience of exercising together with other childhood cancer survivors?
12. Do you have any thoughts about how physical activity affects your body?
13. Do you have any thoughts about the general effects of physical activity?
14. Do you think that there is a need to get advice about physical activity from a physiotherapist because you got an intense treatment during childhood?
15. How are your thoughts about being physically active throughout live?

Question added after the pilot study:

1. Did your family effect your ability to be physical active? If yes, how?
